# Low level phosphorylation of histone H2AX on serine 139 (γH2AX) is not associated with DNA double-strand breaks

**DOI:** 10.18632/oncotarget.10411

**Published:** 2016-07-06

**Authors:** Paulina Rybak, Agnieszka Hoang, Lukasz Bujnowicz, Tytus Bernas, Krzysztof Berniak, Mirosław Zarębski, Zbigniew Darzynkiewicz, Jerzy Dobrucki

**Affiliations:** ^1^ Department of Cell Biophysics, Faculty of Biochemistry, Biophysics and Biotechnology, Jagiellonian University, Krakow, Poland; ^2^ Department of Molecular Biophysics, Faculty of Biochemistry, Biophysics and Biotechnology, Jagiellonian University, Krakow, Poland; ^3^ Laboratory for Imaging of Tissue Structure and Function, Nencki Institute of Experimental Biology PAS, Warsaw, Poland; ^4^ Brander Cancer Research Institute and Department of Pathology, New York Medical College, Valhalla, New York, USA

**Keywords:** DNA damage, topoisomerase inhibitors, transcription, DNA replication, camptothecin

## Abstract

Phosphorylation of histone H2AX on serine 139 (γH2AX) is an early step in cellular response to a DNA double-strand break (DSB). γH2AX foci are generally regarded as markers of DSBs. A growing body of evidence demonstrates, however, that while induction of DSBs always brings about phosphorylation of histone H2AX, the reverse is not true - the presence of γH2AX foci should not be considered an unequivocal marker of DNA double-strand breaks. We studied DNA damage induced in A549 human lung adenocarcinoma cells by topoisomerase type I and II inhibitors (0.2 μM camptothecin, 10 μM etoposide or 0.2 μM mitoxantrone for 1 h), and using 3D high resolution quantitative confocal microscopy, assessed the number, size and the integrated intensity of immunofluorescence signals of individual γH2AX foci induced by these drugs. Also, investigated was spatial association between γH2AX foci and foci of 53BP1, the protein involved in DSB repair, both in relation to DNA replication sites (factories) as revealed by labeling nascent DNA with EdU. Extensive 3D and correlation data analysis demonstrated that γH2AX foci exhibit a wide range of sizes and levels of H2AX phosphorylation, and correlate differently with 53BP1 and DNA replication. This is the first report showing lack of a link between low level phosphorylation γH2AX sites and double-strand DNA breaks in cells exposed to topoisomerase I or II inhibitors. The data are discussed in terms of mechanisms that may be involved in formation of γH2AX sites of different sizes and intensities.

## INTRODUCTION

Phosphorylation of histone H2AX on serine 139 (γH2AX) is an early step in cellular response to DNA damage, when the damage results in formation of a double-strand break (DSB). Regions rich in γH2AX can extend over a stretch as long as 0.5 to 2 Mb, can be asymmetric in relation to a DSB, but do not cover the regions of active transcription [1]. γH2AX foci are readily detected by immunofluorescence microscopy and are generally regarded as markers of DSBs [2, 3]. A growing body of evidence demonstrates, however, that while induction of DSBs always leads to phosphorylation of histone H2AX, the opposite is not the case - the presence of γH2AX foci should not be considered unequivocal evidence of DSBs [4–11]. γH2AX foci exhibit a wide range of sizes and levels of the phosphorylated histone. This fact was generally assumed to reflect no more than an increasing or decreasing extent of H2AX phosphorylation at different time periods elapsing from the induction of a DSB, reflecting the kinetic progression or regression of individual γH2AX foci. Based on the data presented below, we suggest, however, that various sizes and brightness of immunostained γH2AX foci may reflect functional differences. We postulate that some small foci containing low levels of γH2AX may be formed in response to DNA lesions other than DSBs.

Here, we studied DNA damage and γH2AX foci induced by DNA topoisomerase inhibitors. It has been shown previously that camptothecin (CPT), a topoisomerase I (topo1) inhibitor, as well as mitoxantrone (MTX) and etoposide (ETP), topoisomerase II (topo2) inhibitors, induce DNA Damage Response (DDR) in a cell cycle-dependent manner. CPT induces DNA double-strand breaks (DSBs) and γH2AX exclusively in S-phase, at the sites of DNA replication [12–16]. Indeed, it is generally accepted that a collision of DNA replication forks with otherwise “cleavable” complexes of topo1 with DNA, but stabilized by topo1 inhibitor, leads to a collapse of the forks that triggers formation of a DSB [12–14, 16–19]. MTX also induces γH2AX preferentially (although with lesser exclusivity than CPT) in DNA replicating cells, and compared with CPT, with reduced selectivity for the sites of active DNA replication [16, 20]. However, a significant level of histone H2AX phosphorylation is seen also in cells that are not replicating DNA. Interestingly, although the level of γH2AX induced by MTX is significantly higher in S-phase than in nonreplicating cells, γH2AX, and presumably the DNA damage itself, do not necessarily occur in regions of active DNA replication, as we demonstrated previously [16, 20]. ETP presents a different case, in that it induces γH2AX foci in all phases of the cell cycle, with only weak preference to induce damage in DNA of replicating cells or at DNA replication sites [16, 20].

In this study we have turned our attention to three previous observations: (i) γH2AX foci induced by topo1 and topo2 inhibitors in S-phase do not necessarily coincide with sites of DNA replication [16, 20]; (ii) γH2AX foci induced by these drugs exhibit a wide range of sizes and immunofluorescence intensities, and, consequently: (iii) some nuclear foci of H2AX phosphorylation are not spatially associated with 53BP1 (a factor involved in DSB repair [21, 22]) suggesting that they should not be unquestionably regarded as a marker of a DSB [4–11]. Thus, the goal of this work was to understand wheather γH2AX foci formed during an exposure to topoisomerase inhibitors are induced only in response to DSB, or whether they can be formed by other mechanism(s) involving interaction of the inhibitors with chromatin. Our working hypothesis assumed that γH2AX is not exclusively induced by the presence of DSBs. The small γH2AX foci containing low levels of γH2AX, unrelated to DNA replication and observed after treatment with camptothecin or mitoxantrone, are likely to be formed in response to drug-induced distortion of DNA structure or a change in torsional strain of the helix or the presence of single (template) strand DNA breaks formed by RNA polymerases elongating during transcription and colliding with the DNA-bound inhibitor [23]. In the case of DNA replicating cells they may possibly be reflecting DNA replication stress. To investigate this issue we have performed careful analysis of nearest neighbor distances, volumes and fluorescence intensities of foci rich in γH2AX or 53BP1, in relation to DNA replication sites (factories). This approach is based on the assumption that while the presence of γH2AX alone is not a sufficient proof of the existence of a DSB, the concurrent presence of both, γH2AX and 53BP1, the latter a factor thought to promote non-homologous end-joining-mediated (NHEJ) DSB repair, while preventing homologous recombination [24], is a strong indication, if not a sufficient piece of evidence, of a DSB presence in the focus.

We describe here the associations between γH2AX, 53BP1 and replication in subsequent phases of the cell cycle and subphases of S-phase, in a quantitative way, to demonstrate that a low level phosphorylation of histone H2AX on serine 139 is likely induced by stimuli other than DSBs, and is represented by small foci containing fewer γH2AX moieties than the prominent large foci, the latter most likely reporting the presence of DSBs.

## RESULTS

**Figure 1:**
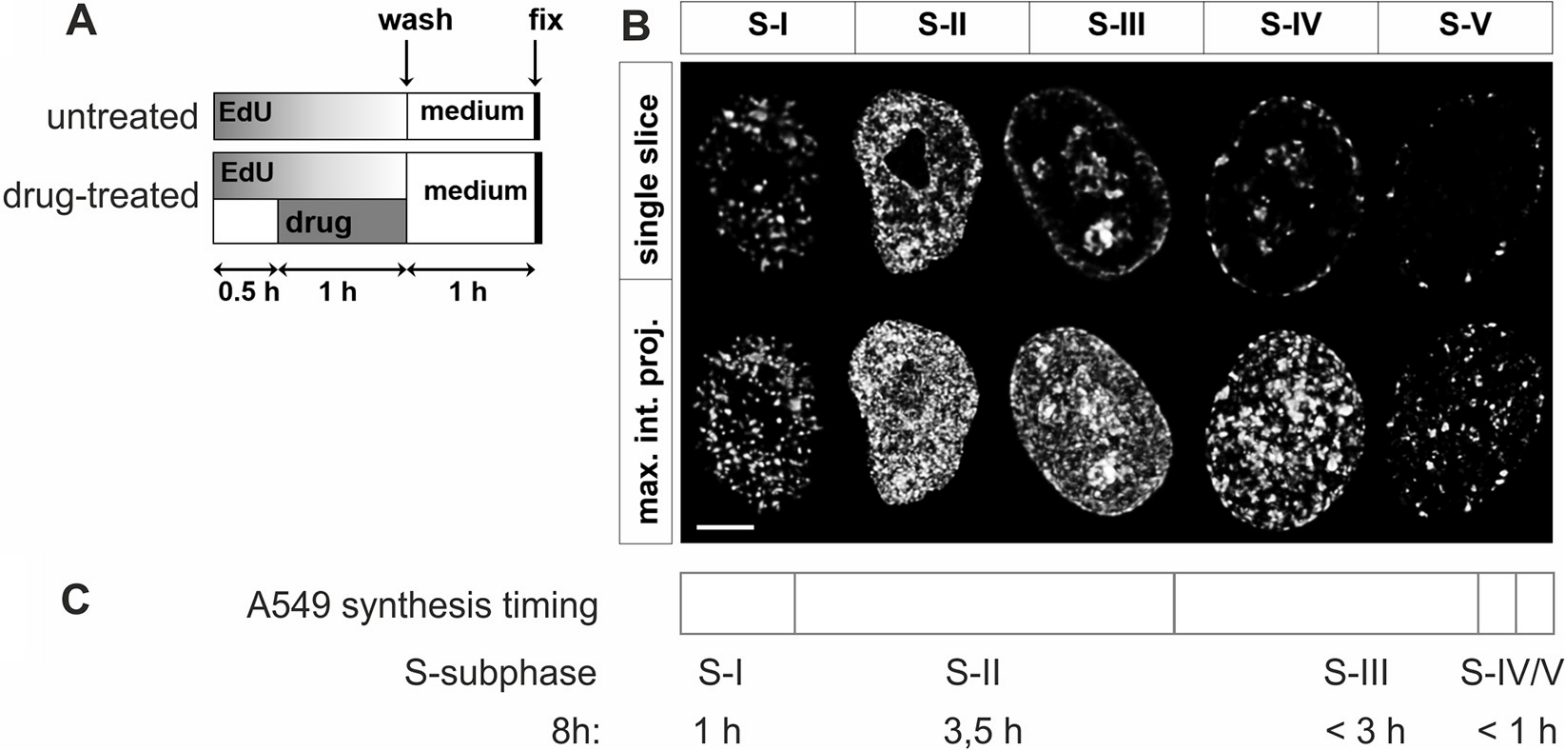
Experimental design and patterns of DNA replication in A549 cells (**A**) Experimental schedule. DNA precursor EdU was added to culture medium at time 0. 30 minutes later a topoisomerase inhibitor was introduced for 60 minutes, still in the presence of EdU. After replacing medium cells were incubated under standard conditions for 1 h to allow histone H2AX phosphorylation, and fixed. (**B**) Confocal images of characteristic patterns of replication factories in subsequent sub-stages of S-phase. S-phase was divided into 5 distinct sub-stages, according to the number and distribution of active replication regions. Note that the duration of these subphases of S varied substantially. Upper row – images of single, equatorial focal planes reveal typical replication patterns, lower row – maximum intensity projections of the whole nucleus (z-stacks); this image representation enables visual estimation of the overall density of fluorescence signals. All images were deconvolved and normalized, therefore, in this form, they do not reflect the total amount of the incorporated EdU. Scale bar 5 μm. (**C**) Timeline of replication in A549 cells, divided into 5 sub-stages, based on live cell imaging of eGFP-PCNA.

**Figure 2:**
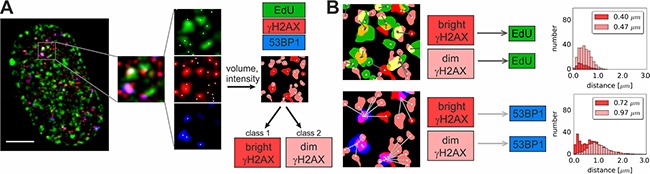
Workflow of 3D data analysis (**A**) Segmentation of fluorescence signals in 3D images. In each detection channel (EdU, γH2AX and 53BP1) coordinates and the number of local intensity maxima representing separate foci are determined. For γH2AX channel the volume and intensity values are computed for each focus in order to classify foci as “dim” or “bright” (see the text for definition). The complete dataset embracing the analysis of volumes and intensities in non-replicating cells and in all sub-stages of S-phase is presented in Supplementary Figure 2. Scale bar 5 μm. (**B**) Nearest neighbor analysis in 3D datasets. The nearest neighbor distances are presented as histograms.

**Figure 3:**
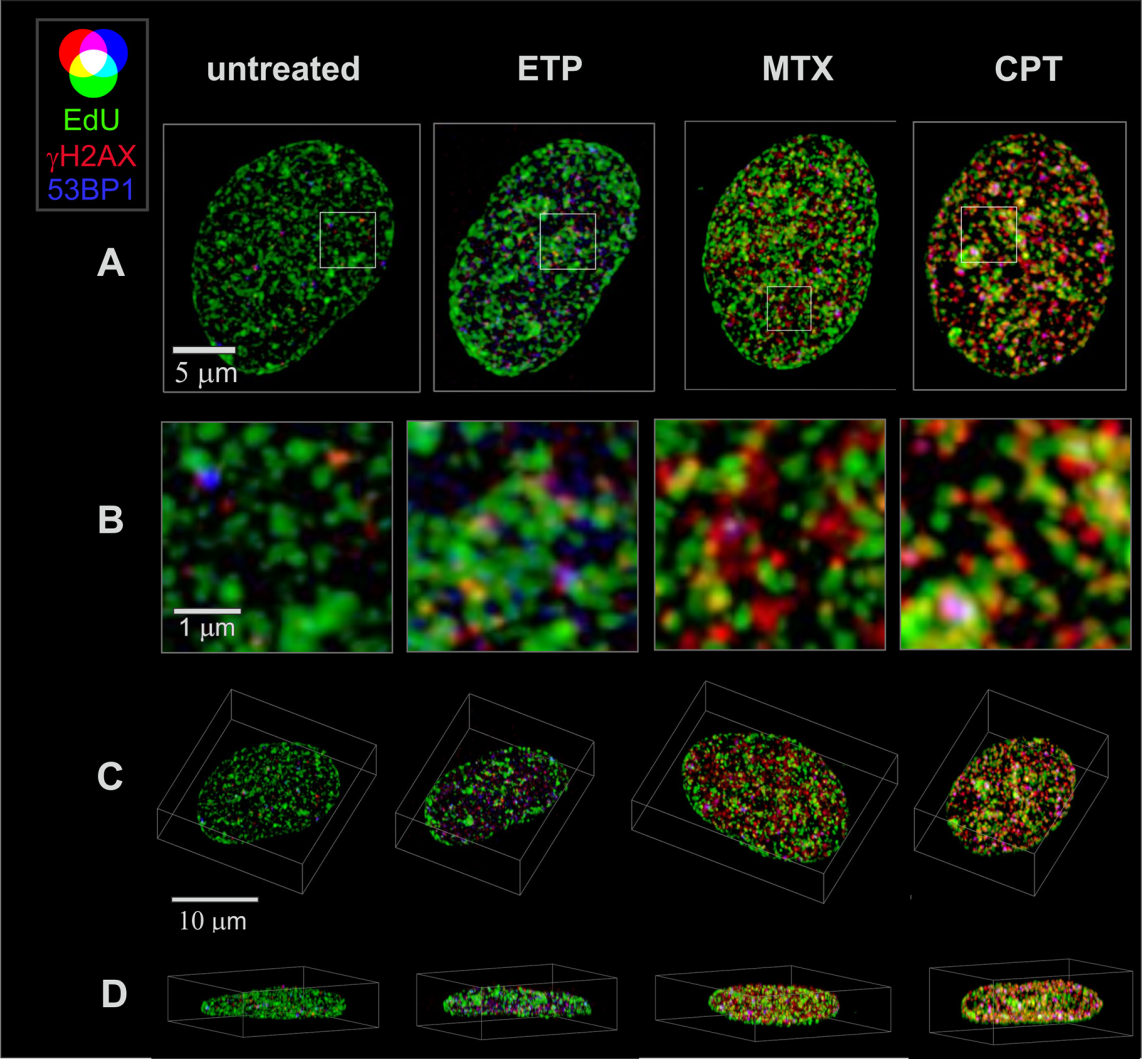
3D images of cell nuclei showing the distribution of replication factories, foci of γH2AX and 53BP1, following exposure to topoisomerase I and II inhibitors Overlapping replication (green - EdU) with DNA damage response (red - γH2AX, blue – 53BP1) regions indicates selectivity of DNA damage induction towards active regions of replication. Maximum intensity z-projections of confocal stacks following deconvolution and normalization (**A**), enlarged selected areas (**B**), 3D maximum intensity xyz images (**C**), and xz (**D**) projections are shown.

**Figure 4:**
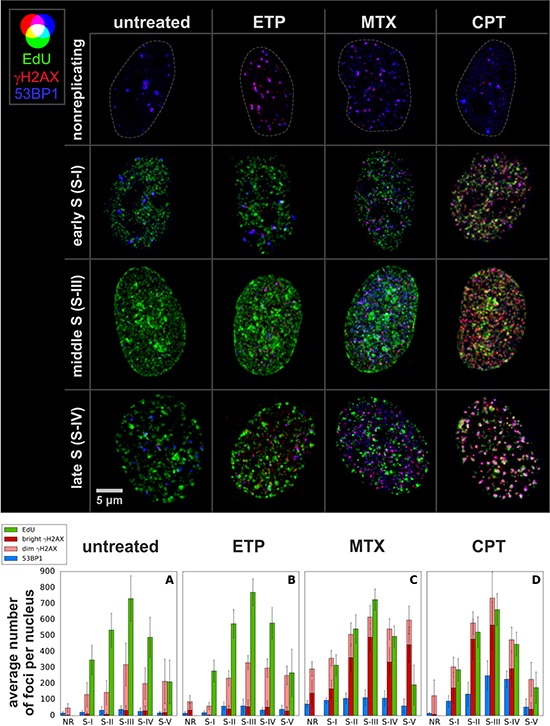
Images of nuclei (max. int. z projections) in early, mid and late S, and the average numbers of replication (EdU) and DDR (γH2AX, 53BP1) foci in cells exposed to topoisomerase inhibitors Each bar is based on determination of foci numbers in ca. 20 nuclei. Note, that in cells that suffered heavy damage the density of γH2AX foci is high, therefore the number of “dim” γH2AX foci is underrepresented. A complete statistical analysis of the numbers of foci is included in Supplementary Figure 3.

**Figure 5:**
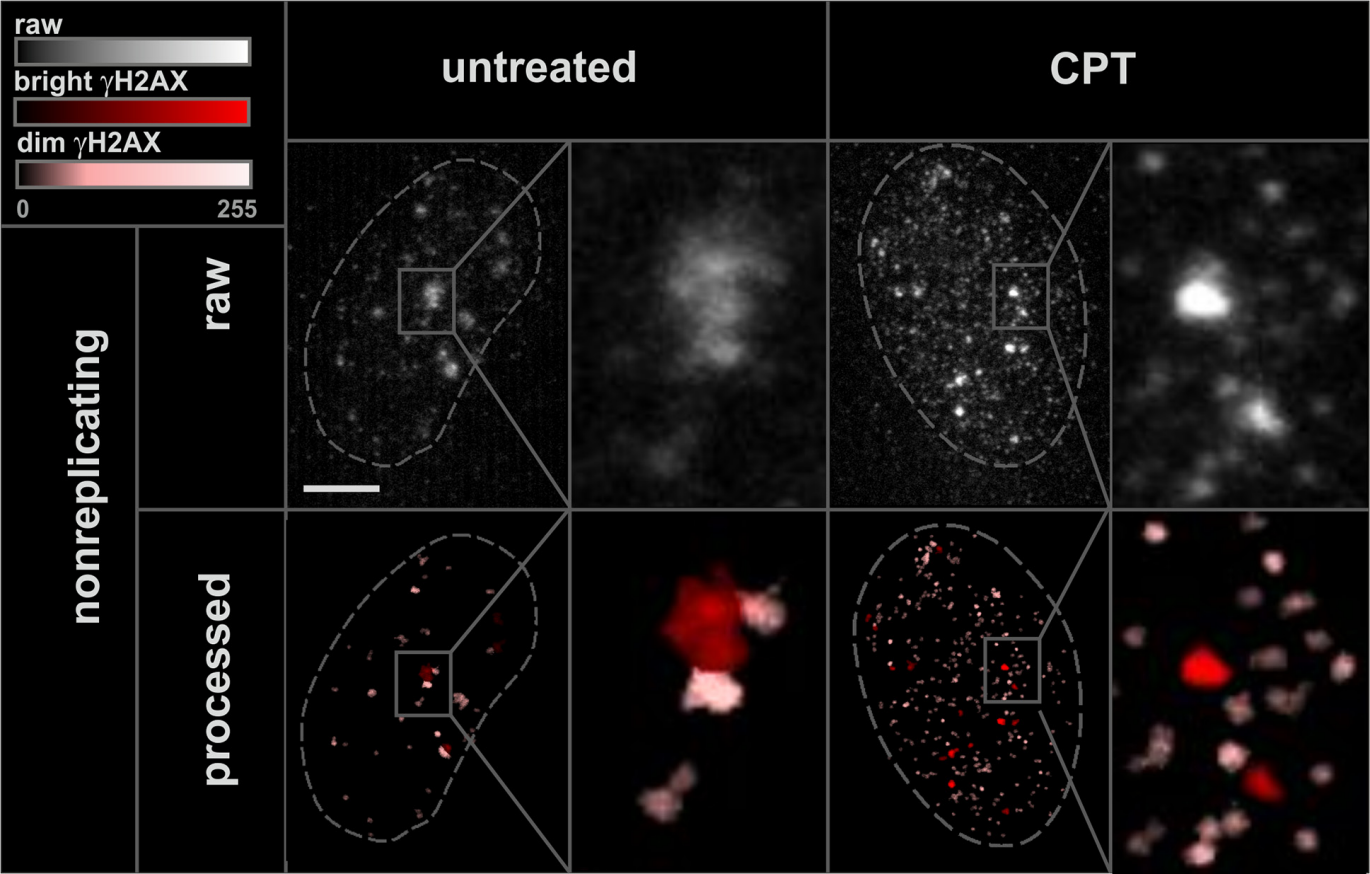
“Bright” and “dim” γH2AX foci Maximum intensity projection images, and histograms of raw 3D image stacks (enhanced by increasing the brightness), and the processed images of the same areas, displayed by using color-coded classification for “bright” and “dim” γH2AX.

In order to study γH2AX foci of various size and brightness we exposed A549 cells to CPT, MTX or ETP according to a protocol described in Figure 1A, and imaged foci of γH2AX, 53BP1 as well as DNA replication factories in cells in various sub-stages of S-phase (Figure 1B, 1C). The workflow of analysis is described in Figure 2. The number of γH2AX foci identified in cells incubated with each of the studied topoisomerase inhibitors was far greater than in untreated cells. Maximum z-projection images and 3D reconstructions, shown in Figure 3, convey the information about the overall density of foci of the three types, while the central cross-sections, shown in Supplementary Figure 1, reveal more clearly the characteristic patterns of replication foci in subsequent sub-stages of S-phase. The actual numbers of foci are given in Figure 4 and Supplementary Figure 3 (for all substages of S-phase; the particular substages were identified based on a characteristic pattern of spatial location of DNA replication sites, as described before [25, 26]). There was a great variation in sizes of the immunostained phosphorylated regions of chromatin, and a wide range of fluorescence brightness, a parameter, which is proportional to the amount of γH2AX in each focus (Supplementary Figure 2). In order to understand the link between DSBs and histone H2AX phosphorylation, in the context of DNA replication and the damage caused by topoisomerase inhibitors, we analyzed the positions of γH2AX foci in relation to the foci of 53BP1 and DNA replication sites. The data analysis entailed subdivision of γH2AX foci into two classes, as shown schematically in Figure 2A. The first class (“bright” foci) embraced foci of a large size or brightness (at least 0.25 μm^3^ volume or at least 35 gray levels in 8-bit scale mean fluorescence intensity images), while the second (“dim” foci) included small size and low brightness foci (less than 0.25 μm^3^ volume and less than 35 gray levels). Figure 5 shows an example of a maximum intensity projection of a raw image of γH2AX and the assignment of foci to the respective classes, in untreated and CPT-treated cells. We have analyzed the positions of γH2AX foci in relation to regions of replication and foci of 53BP1, as described below.

### Endogenous (constitutive) H2AX phosphorylation

**Figure 6:**
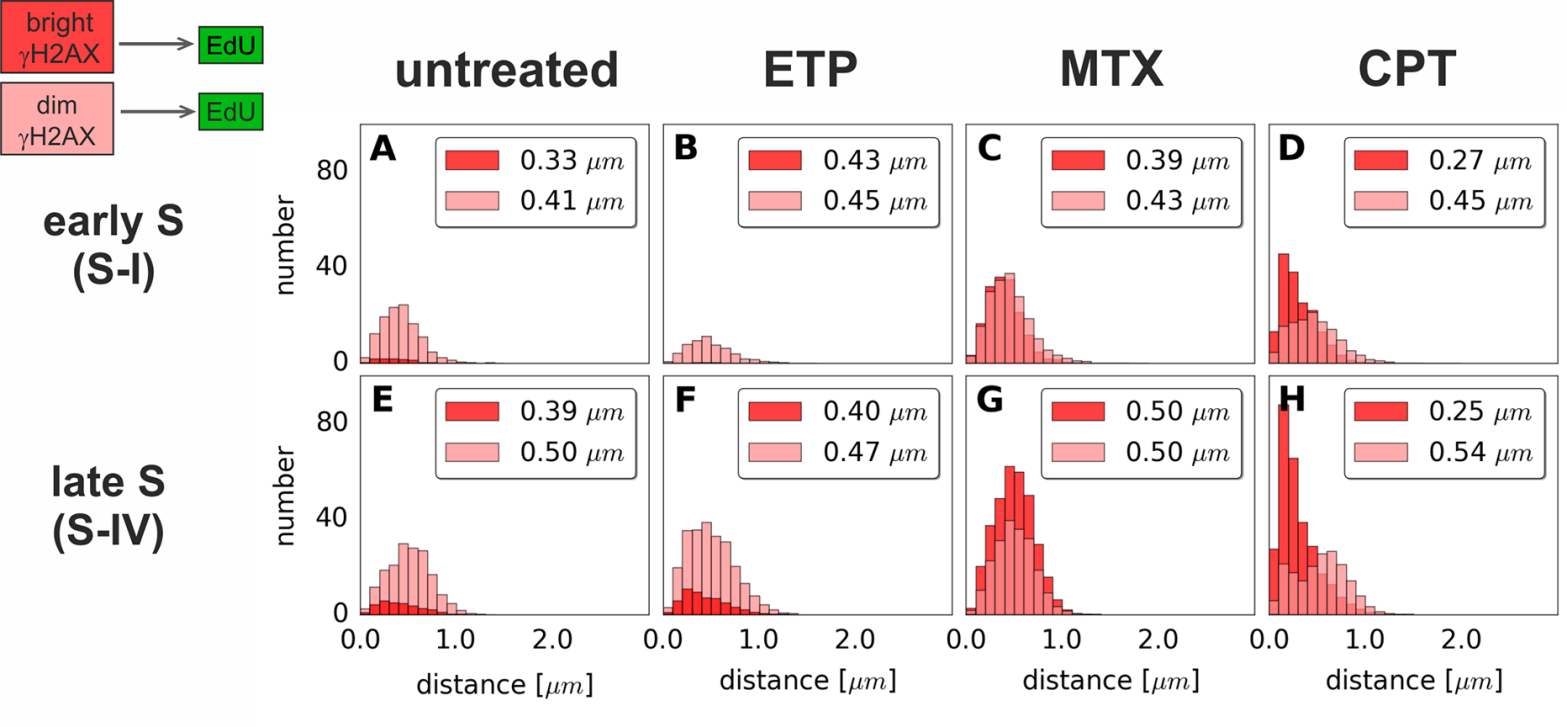
Histograms of distances from “bright” or “dim” γH2AX foci to the nearest region of replication (EdU) Distances from each γH2AX focus of either class (“bright” or “dim”) to the nearest replication (EdU) site have been measured. Data for foci in very early S (S-I, replication of euchromatin) and late S (S-IV, replication of heterochromatin) are shown. The median value of the distance is given in the upper right corner of each panel. Each histogram represents the averaged data based on at least 20 nuclei per class. The complete analysis of all sub-stages of S-phase is given in Supplementary Figure 4.

**Figure 7:**
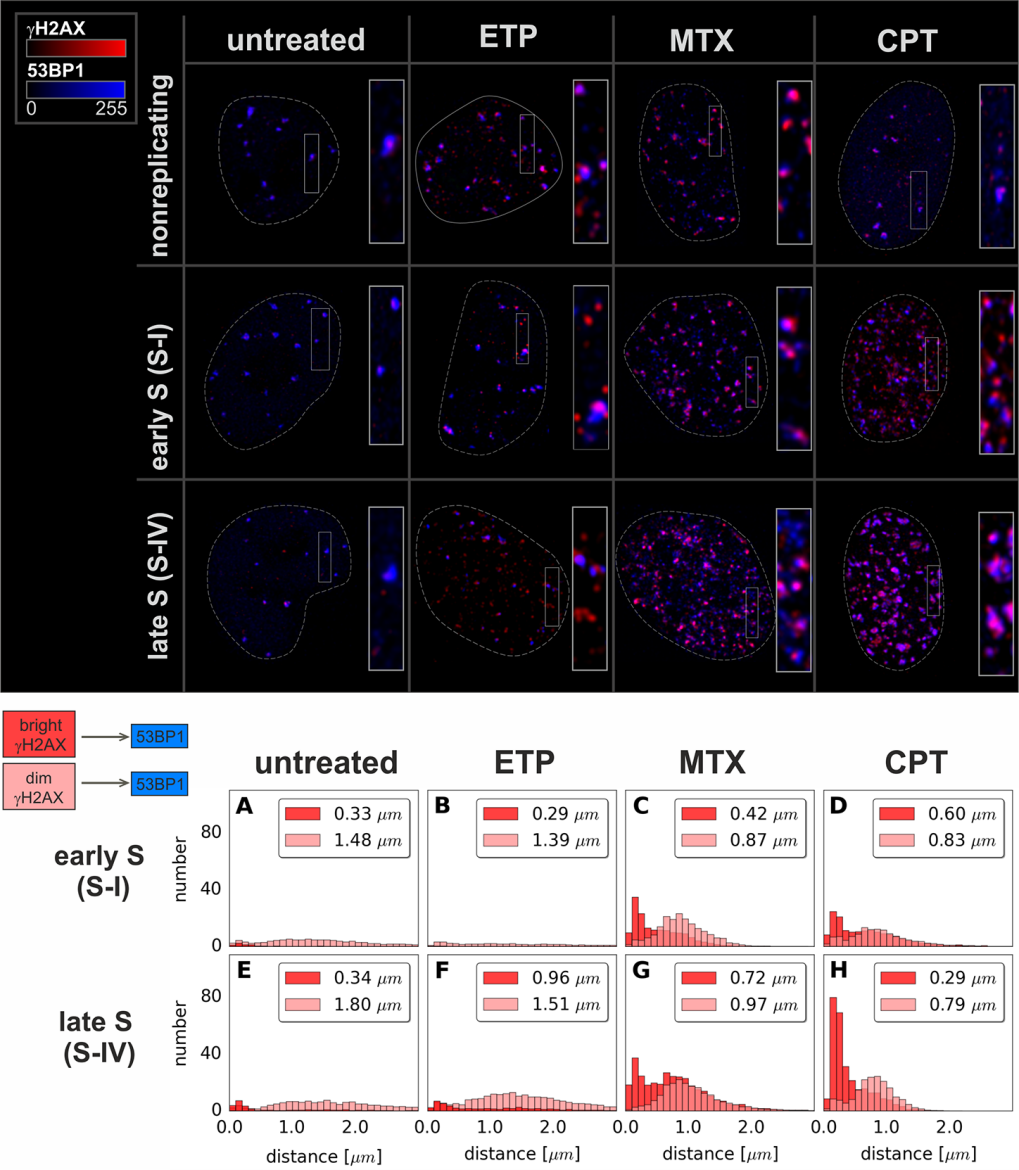
Maximum intensity projection images and histograms of distances from “bright” or “dim” γH2AX foci to the nearest 53BP1 focus Data for foci in very early S (S-I, replication of euchromatin) and late S (S-IV, replication of heterochromatin) are shown. The median value of the distance is given in the upper right corner of each panel. Each histogram represents the averaged data based on at least 20 nuclei per sub-stage of S-phase. Complete analysis corresponding to every sub-stage of S-phase is given in Supplementary Figure 5.

We first analyzed ‘bright’ γH2AX foci in untreated cells, thought to report predominantly DNA damage induced by endogenous oxidants [27]. In untreated S-phase cells the number of “bright” γH2AX foci correlated weakly with the number of DNA replication sites (Figure 4A, Supplementary Figure 3A). The histograms of distances between “bright” γH2AX foci and replication factories (Figures 6A, 6E, and Supplementary Figure 4 (untreated)) demonstrated that there was no definite preference for γH2AX foci to occur at an immediate vicinity of replicating DNA. Almost all “bright” γH2AX foci had a closely associated 53BP1 focus, suggesting their presence at the sites of DSBs (Figures 7A, 7E, and Supplementary Figure 5 (untreated)). These observations imply that the “bright” γH2AX foci in untreated cells represent DSBs. However, the shape of the histogram (Figure 7A, 7E) and the correlation analysis (Supplementary Figure 7 (untreated)) indicate that there might be a minor tendency of γH2AX foci to be associated with DNA replication. Thus, the probability of induction of DSB by endogenous factors in replicating regions are only slightly higher than in the sites of DNA that do not undergo replication. This assessment agrees with our previous observations [16].

The number of “dim” γH2AX foci in untreated cells was far greater than the “bright” ones (Figure 4A, Supplementary Figure 3A). In the case of DNA replicating cells their number approximately correlated with the number of DNA replication sites. However, there was no apparent proximity between these sites in 3D space. Specifically, the ‘dim” γH2AX foci, although very numerous in S-phase, appeared not to be preferentially located at-, or near- replicating DNA (Figures 6A, 6E, Supplementary Figure 3, and Supplementary Figure 4 (untreated)). They were not associated with 53BP1 either (Figures 7A, 7E, Supplementary Figure 3 and Supplementary Figure 5 (untreated)). We conclude that the “dim” γH2AX do not represent DSBs, and may be marking other type of DNA lesions.

### Histone H2AX phosphorylation induced by topoisomerase inhibitors - the “bright” γH2AX foci

Phosphorylation of H2AX foci induced by the three topoisomerase inhibitors varied in size and brightness (Supplementary Figure 2). The “bright” and the “dim” foci were analyzed separately.

### Camptothecin

The numbers of “bright” γH2AX foci induced by CPT correlated with the numbers of DNA replication foci (Figure 4D, Supplementary Figure 3D) and were significantly greater in S-phase than in the cells not replicating DNA (Supplementary Figure 2 (CPT) and 3D). The “bright” γH2AX foci were located in a close proximity to DNA replication factories (Figure 6D, 6H and Supplementary Figure 4 (CPT)) and a large proportion of these foci (especially in late S-phase) were associated with 53BP1 (Figure 7H, images “CPT” and Supplementary Figure 5 (CPT)). Therefore these foci were most likely representing DSBs. We hypothesize that a subpopulation of “bright” γH2AX foci that were not associated with 53BP1 represented the sites of newly induced DSBs, where the repair process involving recruitment of 53BP1 did not yet commence at the time of cell fixation. These observations are in agreement with the already published data that CPT induces DSBs and DDR in replicating regions of DNA [16]. We conclude that a very large proportion, if not all “bright” γH2AX foci detected in S-phase in CPT-treated cells represent DSBs.

### Mitoxantrone

MTX also induced “bright” γH2AX foci (Supplementary Figure 3 (MTX)), and their number correlated with the number of replication factories, especially in early and mid S-phase (Figure 4C, Supplementary Figure 3C). However there was no obvious preference for “bright” γH2AX foci to be located in or near DNA replication sites (Figure 6C, 6G). A large subpopulation of γH2AX foci were located within or adjacent to 53BP1 (Figure 7C, 7G, and Supplementary Figure 5 (MTX)), suggesting that these foci corresponded to DSBs. As in the case of CPT, however, there was also a large number of γH2AX foci not accompanied by 53BP1. In summary, although MTX showed a propensity to induce DSBs in S-phase cells, and caused formation of large γH2AX foci, unlike CPT, these lesions were not linked directly to DNA replication sites.

### Etoposide

The number of “bright” γH2AX foci in the cells treated with ETP was relatively low (Figure 4B, Supplementary Figure 3B, Supplementary Figure 2 (ETP)). Most of these foci correlated with 53BP1 (Figure 7B, 7F and Supplementary Figure 5 (ETP)) but not with DNA replication sites (Figure 6B, 6F and Supplementary Figure 4 (ETP)). This is an indication that they represented DSBs, but with no direct link to replication. Most of the γH2AX signal induced by ETP was associated with the “dim” foci that were almost entirely located at a distance from DNA replication regions, as discussed below.

### Histone H2AX phosphorylation induced by topoisomerase inhibitors - the “dim” γH2AX foci

Interestingly the analysis of the “dim” and “bright” γH2AX foci induced during exposure to topoisomerase inhibitors demonstrated that, in general, the positions of these foci were different in relation to replication factories and 53BP1 foci (Figure 6, Figure 7). Specifically, unlike the “bright” foci, the “dim” γH2AX foci revealed no clear association, with 53BP1 or with DNA replication.

### Camptothecin

The most conspicuous case of “dim” γH2AX foci is presented by exposure to CPT. Here the “dim” γH2AX foci were numerous but exhibited neither proximity to replication sites nor to 53BP1 foci (Figure 6D, 6H, Supplementary Figure 4 (CPT), Figure 7D, 7H and Supplementary Figure 5 (CPT), respectively). Only a very small proportion of the “dim” foci was associated with 53BP1, suggesting that these particular foci may have been in an early stage of histone H2AX phosphorylation induced by a DSB. However, the majority of “dim” γH2AX foci were not associated with 53BP1 or replication, suggesting that they did not represent DSBs. Since the mechanism of DNA damage induced by CPT in DNA undergoing replication is known in detail [17, 18, 28], and we are not aware of any evidence of CPT inducing DSBs that would be marked by γH2AX outside of the replicating DNA, the presence of “dim” γH2AX in regions distant from replication and devoid of 53BP1 speaks in favor of a notion that the low level H2AX phosphorylation is unrelated to the presence of DSBs. This statement is corroborated by the fact that we observed a statistically significant increase of “dim” γH2AX foci when non-replicating A549 cells were challenged with CPT (Figure 4D, nonreplicating cells and Supplementary Figure 2D). We have seen a similar phenomenon in HeLa cells and in normal human fibroblasts derived from healthy donors and grown in culture (data not shown). Thus, the available evidence suggests that these γH2AX signals must have been induced by mechanism(s) other than formation of DSBs.

It is important to note that the number of detectable “dim” γH2AX foci was generally lower in replicating cells treated with CPT or MTX, and higher in replicating cells exposed to ETP, or in the untreated controls. This difference is, at least in part, due to the fact that “bright” γH2AX foci in DNA replicating cells are very numerous after exposure to CPT or MTX, therefore their 3D images occupy a large proportion of the available space and overshadow the small “dim” foci. Thus, because of this hindrance, in samples with a high number of “bright” γH2AX foci, the number of “dim” foci was underestimated. Since the density of foci of various classes influences the probability of their accidental overlap including the mean distances between foci of two types, and, consequently influences the shapes of the histograms of these distances, we have also preformed correlation analysis, using the approach, which was mathematically defined in detail previously [20].

Analysis of normalized autocorrelation functions of the “bright” γH2AX foci revealed their non-random clustering (L > 0) in cells treated with CPT and ETP, at distances shorter than 1 μm (Supplementary Figure 6F and 6H, respectively). This effect is most prominent in late S (ETP: S-IV yellow line, ETP and CPT: S-V red line). On the other hand, no clusters were observed in cells treated with MTX (Supplementary Figure 6G). In untreated cells constitutive (endogenous) “bright” γH2AX foci tended to form clusters in all subphases, since the primary sites of damage were DNA replication foci containing multiple active replication forks (Supplementary Figure 6E). Note, that the number of foci analyzed in untreated cells was very low. Moreover, the clustering of “bright” γH2AX foci in the control and the CPT or ETP treated cells was independent of the distribution of DNA replication sites. Thus, it can be speculated that the grouping of “bright” γH2AX foci was not determined by this process. It may also be noted that a similar pattern of clustering was detectable in the case of 53BP1 foci (Supplementary Figure 6M–6P). Regardless of the treatment and the sub-stage of S-phase autocorrelation (L) function values of “dim” γH2AX foci were close to 0 (at all distances), which indicated their uniform distribution.

Analysis of normalized cross-correlation (L) function revealed that, in cells treated with CPT, replication and “bright” γH2AX foci coincided at distances shorter than 0.5 μm (Supplementary Figure 7D). This effect was reflected in the values of L-function being larger than 0 (random correlation) and was most prominent in the late S-phase. In control cells the correlation was weaker, but clearly detectable in the late S. In the case of cells treated with ETP and MTX, correlation between CPT “bright” γH2AX foci was less pronounced and detectable (i.e. non-random) at distances shorter than 0.3 μm. This is likely to reflect a non-uniform distribution of nuclear chromatin density, while the calculations of random correlation values were based on the assumption of uniformity (see Materials and Methods). Likewise, a strong correlation between the “bright” γH2AX foci and 53BP1 at distances shorter than 0.5 μm was detectable in cells treated with CPT, ETP and in the untreated cells (Supplementary Figure 7I–7L). This correlation was less pronounced in the cells treated with MTX, though.

No significant correlation between replication regions and “dim” γH2AX foci was observed in cells treated with topoisomerase II inhibitors (ETP and MTX) and in control (Supplementary Figure 7E–7G). On the other hand some degree of correlation between these signals was detected (particularly in S-IV subphase) in cells treated with CPT (Supplementary Figure 7H). This notion is compatible with the results of the nearest neighbor analysis. One may postulate that, in contrast to the effects of ETP and MTX, the population of “dim” γH2AX foci (CPT) was heterogeneous and some of the foci correspond to replication-associated DNA lesions. Likewise, in the treated cells correlation between the “dim” γH2AX foci and 53BP1 foci was slightly stronger in the case of CPT than in the case of ETP and MTX (Figure 7N–7P). Nonetheless, in all these cases the strength of the “dim” γH2AX foci correlation was significantly lower than the respective value calculated for their “bright” counterparts. In summary, we conclude that correlation analysis confirmed the association between the “bright” γH2AX foci and DNA replication sites and 53BP1 foci, and a lack of such an association between the “dim” foci and DNA replication (Supplementary Figure 6 and Supplementary Figure 7).

### Mitoxantrone

The number of “dim” γH2AX foci in MTX treated cells correlated with the number of replication foci and was similar (in early S) or lower (in mid and late S) than the number of “bright” foci (Figure 4C, Supplementary Figure 3C). The same rule applied – the “dim” foci were not located close to the replication factories (Figure 6C, 6G and Supplementary Figure 4 (MTX)), nor did they have 53BP1 associated with them (Figure 7C, 7G and Supplementary Figure 5 (MTX)). This observation, again, supports the notion that the “dim” γH2AX foci were unrelated to DSBs.

### Etoposide

In the case of ETP the number of “dim” γH2AX foci was much greater than of “bright” foci (Figure 4B, Supplementary Figure 3B) and the “dim” foci did not coincide with replication factories (Figure 6B, 6F and Supplementary Figure 7 (ETP)). While only very few ETP-induced “dim” γH2AX foci were associated with 53BP1 most of them were located afar of 53BP1 (Figure 7B, 7F, and Supplementary Figure 5 (ETP)), suggesting that this widespread but weak phosphorylation of histone H2AX was not representing DSBs.

## DISCUSSION

It is now generally accepted that mammalian cells respond to induction of DSBs by phosphorylation of histone H2AX in nucleosomes located over long stretches of DNA on both flanks of the break [1–3]. It appears that despite occasional exceptions, a reverse statement has also been well entrenched by now – that of γH2AX being a specific marker for DSBs. However, several reports demonstrated that γH2AX may also occur in chromatin regions that do not harbor DSBs marked with 53BP1, and can be induced by hydrogen peroxide [11], UV irradiation [9], or other stimuli [29]. Such γH2AX foci were observed in senescent cells [4], mitotic cells [7] and in mouse embryos [8]. Interestingly, it has also been demonstrated that phosphorylation of H2AX can be initiated by a stimulus, which is not necessarily a damage event itself [30]. We also note, however, that although in several reported cases γH2AX was not directly linked to DSBs, the presence of such DNA breaks could not be ruled out, as discussed in [31].

The fact that γH2AX foci detected by immunofluorescence come in a variety of sizes and intensities was often explained as due to kinetics of their regression as a function of time following their induction by the DSB. Here we present quantitative analysis of sizes of these foci, the levels of phosphorylation, position relative to foci containing 53BP1 (a protein, which is involved in DSB repair), and DNA replication sites to substantiate our assertion that γH2AX foci that are small in size and contain low levels of the phosphorylated histone H2AX are formed in response to DNA damage or stress but not the presence of DSBs.

As this notion contradicts the generally established (even if questioned already [4–11]) opinion, the most likely interpretation of the presence of “dim” γH2AX foci is likely to center on a suspicion that they simply represent either a time-related regression of the large bright foci, a technical artifact, certain kind of nonspecific staining, or noise. Indeed, in our previous work we (and presumably other labs interested in γH2AX imaging) assumed that low level of phosphorylation or small (‘dim’) γH2AX foci should be classified as a “background” or a certain kind of staining of unspecified mechanism. A known molecular mechanism of DSB induction by camptothecin [12–14], a priori knowledge regarding the induction of DNA strand breaks by topoisomerase inhibitors detected by the TUNEL assay [32], careful optimization of labeling procedures and quantitative analysis, as done in the present study, lead us to propose, that these ‘dim’ foci may have a possible functional significance, although of a still unidentified nature.

Low intensity γH2AX immunofluorescence may adopt a form of a relatively uniform level of evenly distributed staining or small foci characterized by fluorescence levels only slightly exceeding the noise level. Their appearance is dependent on the quality of immunofluorescence staining, the type of the objective lens used, and the image processing techniques leading to a final image. We note the fact that several nuclear proteins we studied look as uniformly distributed in a live cell image of an FP-tagged fusion protein, but appear as grainy or dotty after fixation and immunofluorescence detection of the endogenous or the fusion protein. Even if the subnuclear distribution of γH2AX (more uniform or more foci-like) is contested, this does not alter the final conclusion of the reasoning presented here. Whether the low level phosphorylation is induced in a form of small foci or of somewhat larger sites, in both cases we do not observe any association with 53BP1 or DNA replication factories. If γH2AX were associated with DSBs only, such an association should be present, and it should be close to a 1-to-1 relationship. A high correlation between γH2AX and 53BP1 is found only in the case of “bright”, but not the “dim” foci γH2AX foci in CPT-treated cells in S-phase.

Further arguments in favor of the notion that the minor phosphorylation sites do not constitute a nonspecific background, a technical artifact, or a phenomenon related to DSBs, are based on the following observations:

1. The number of “dim” γH2AX foci increases as cells move from G_1_ into S-phase in control, untreated cells. The signal representing nonspecific binding of the secondary antibody, which is used to detect γH2AX, is not expected to depend on the level of replication,
2. There is a statistically significant higher number of “dim” γH2AX foci in CPT-treated cells that do not replicate DNA, than in untreated ones. CPT is not expected to induce DSB in cells not replicating DNA, thus the “dim” γH2AX foci in such cells can reasonably be interpreted as a chromatin modification unrelated to DSBs, yet still associated with a CPT insult,
3. The majority of “dim” γH2AX foci are not located close to 53BP1 or DNA replication sites, while the “bright” γH2AX foci are mostly associated with DNA replication (in CPT or MTX treated cells). If “dim” γH2AX were just representing an early stage of phosphorylation at DSB sites, 53BP1 would be expected to be present in (at least) some of these sites as well. No such correlation was seen,
4. If the ‘dim” γH2AX foci were marking DSBs induced at the sites of DNA replication in a very early stage of S-phase, incorporation of some EdU would be detected at these sites. We, as well as others, have shown that EdU is a sensitive marker of replication, with no detectable nonspecific staining or background [33]. Our microscopy system is capable of detecting DNA replication with high sensitivity, therefore the rate of false negative identification of S-phase cells is very low if any. No EdU signals associated with “dim” γH2AX were detected.

The arguments presented above essentially exclude the possibility that the “dim” foci are the markers of DSBs. The question may be asked therefore what kind of DNA or chromatin lesions may generate them. The following events may be accountable for it:

### Untreated cells

It is likely that in the case of the untreated, control cells the reactive oxygen species (ROS) generated during oxidative phosphorylation are responsible for the “constitutive H2AX phosphorylation” as described by us before [27]. Consistent with the current findings we observed then an increased intensity of γH2AX immunofluorescence in the S-phase cells as compared with G_1_ cells. γH2AX immunofluorescence of these cells was decreased by over 50% by the short (1 h) cells exposure to the classical ROS scavenger N-acetyl-L-cysteine [27].

### CPT-treated cells

CPT binds to both topo1 and DNA with hydrogen bonds stabilizing, the otherwise transient, covalent topo1-DNA complex [34, 35]. This prevents DNA re-ligation and therefore causes DNA damage that results in apoptosis. The latter however occurs only when DNA replication forks collide with the stabilized complexes [18]. However, because we did not see the spatial association between the EdU incorporation and the “dim” foci it is likely that just stabilization of the covalent topo 1 and DNA complexes may be recognized by the cell as the lesion that can trigger H2AX phosphorylation leading to formation of the “dim” foci.

### MTX-treated cells

The anthracycline antibiotic MTX, binds to DNA by intercalation elongating the ds DNA helical structure [36, 37]. Furthermore, it also has an ability to condense nucleic acids, including DNA, with an affinity related to DNA bases composition [38]. Thus, in the case of cells not replicating DNA these modes of deformation of the double helix may lead to an induction of H2AX phosphorylation in form of the “dim” foci. It should also be noted that although MTX was introduced to substitute its analogue doxorubicin because of toxicity (cardiotoxicity) of the latter related due to induction of oxidative stress, although being much less toxic, MTX also generates ROS after binding to cells [39].

### ETP-treated cells

ETP inhibits the re-ligation reaction of topo2 after it nicks the two strands of DNA, trapping it in a cleavable complex consisting of two topo2 subunits covalently linked to the 5′ ends of DNA. Because the two subunits interact strongly with each other to hold the two ends of DNA together this structure is not directly recognized as a true DSB by cells [40–42]. Furthermore ETP was shown to have higher affinity to chromatin compared to DNA and that the globular domain of histones was the site of its binding [43]. This binding of ETP to histones was shown to alter their secondary structure accompanied with hypochromicity revealing compaction of histones in the presence of the drug [43]. The both types of interaction of ETP, either with DNA and/or with chromatin, may be responsible for the presently observed induction of the “dim” γH2AX foci.

Needless to say, the constitutive phosphorylation of H2AX as reported in the case of untreated, control cells and related to the production of oxidative species by mitochondria [27] and presently seen in our untreated cells, likely contributed to the formation of “dim” γH2AX foci in the CPT-, MTX- as well ETP- treated cells.

It should be noted that in addition to the effect of topoisomerase inhibitors on DNA replication, as discussed above, also their impact on transcription may play a role in inducing H2AX phosphorylation that can lead to the “dim” foci. It was shown that DNA breaks resulting from collision of elongating RNA polymerase with the inhibitor-DNA binding sites take place only on a single (template) DNA strand whereas the non-template strand within the transcription bubble remains intact [23, 44]. It is likely therefore, that the “dim” γH2AX foci observed by us may also mark the ssDNA breaks occurring as a consequence of a collision of elongating RNA polymerase with the DNA-inhibitor sites [28]. This mechanism is consistent with the observation that the “dim” foci were seen in the cells treated with the DNA binding topoisomerase inhibitors CPT or MTX but not in the cells treated with the topo2-binding ETP. In support of this mechanism are also observations that short treatment of A549 cells with 2 or 20 nM MTX dramatically decreases the rate of transcription, an evidence of formation of MTX-DNA complexes that stop progression of RNA polymerase [44].

## MATERIALS AND METHODS

### Cell cultures and drug treatment

A549 human lung adenocarcinoma cells were obtained from ATCC and maintained as described previously [26]. Exposures to the drugs were commenced 24–48 h after seeding, when cells were in the exponential phase of growth and reached approximately 70% confluency. In order to fluorescently label nascent DNA synthesized prior to an exposure to a topoisomerase inhibitor, a DNA precursor (EdU, 10 μM, Molecular Probes/Thermo Fisher Scientific) was added to culture medium 30 minutes before exposure to the drug [45]. The drug exposure lasted for 1 h, in the presence of EdU (0.2 μM CPT, Sigma-Aldrich; 10 μM ETP, Sigma-Aldrich; or 0.2 μM MTX, Hospira). After the drug treatment culture medium was removed, cells were washed twice with a pre-warmed PBS, and incubated in fresh culture medium for 1 h. Subsequently cells were washed with PBS again and fixed with formaldehyde (4%, methanol free, at RT, for 15 min, Electron Microscopy Sciences (Figure 1A)).

### Staining procedure

Before immuno-labeling, cells were permeabilized with 0.5% Triton-X 100 and blocked overnight with BSA (3%, w/v) in moist chamber in 4°C. DNA damage response and repair of double strand breaks (DSB) were detected by fluorescence immunostaining of γH2AX [2] and of 53BP1. A mixture of primary antibodies was applied for 1 h, at RT (phospho-specific γH2AX mAb - 1:350 dilution, Millipore, cat no. 05-636; 53BP1 IgG - 1:200 dilution, Abcam, cat. no. ab21083). After washing, cells were incubated for 1 h with a mixture of secondary antibodies (goat anti-mouse IgG (H+L) AlexaFluor555 – 1:1000, Molecular Probes/Thermo Fisher Scientific, cat. no. B00079; and goat anti-rabbit IgG (H+L) and 1:2000 dilution of AlexaFluor488 – 1:2000, Molecular Probes/Thermo Fisher Scientific, cat. no. B00072). After each incubation cells were washed gently with PBS with Ca^2+^ and Mg^2+^ for at least 1 h with 7 or more replacements of the washing solution. Care was taken not to allow the sample to dry.

In order to label newly replicated chromatin, incorporation of EdU, followed by ‘click’ reaction with a fluorescent label was performed (Click-iT^®^ EdU AlexaFluor647N Imaging Kit; Molecular Probes/ Thermo Fisher Scientific). The labeling procedure was carried out according to the manufacturer instructions. After labeling, samples were mounted on glass slides using ProLong Gold Antifade Mountant (Molecular Probes/Thermo Fisher Scientific).

### Confocal imaging

Leica TCS SP5 confocal system (Leica Microsystems GmbH, Wetzlar, Germany) was used to image fixed cells. The following instrumental parameters were used: 63× HCX PL APO CS NA 1.4 oil immersion lens, confocal iris set at 1 Airy disc, excitation 488 nm (Ar laser), 561 nm (DPSS laser) and 633 nm (HeNe laser), emission detection bands 500–550 nm for AlexaFluor488 (immunofluorescence of 53BP1), 570–620 nm for AlexaFluor555 (immunofluorescence of γH2AX) and 660–720 nm for AlexaFluor647N (EdU Click-iT detection). Registration was performed in sequential mode, with a scanning rate of 8000 Hz (resonant scanner), 8 bit dynamic range, with 16 averaged frames for one confocal plane. One 3D stack consisted of at least 80 confocal slices, confocal planes (512 × 512 pixels, pixel size 60 nm) were separated by 130 nm along z axis.

### Image processing and quantitative image analysis

3D images were deconvolved using Huygens Deconvolution & Analysis Software (Scientific Volume Imaging B.V., Hilversum, Netherlands). Quantitative analysis of the deconvolved images representing replication factories or γH2AX or 53BP1 foci was used to determine their position, number and volume. Raw images were used to estimate mean fluorescence intensity of individual γH2AX foci.

The analysis was carried out with the use of algorithms developed under ImageJ macro language and Python. Prior to estimation of foci position, deconvolved stacks were corrected for registration shift and oversaturated pixels. Local fluorescence maxima within foci were designated using 3D max finder software [16]. Briefly, coordinates were calculated as the conjunction of two individual searches in orthogonal sections with the use of ImageJ plugin ‘Find Maxima’. Search parameters based on foci density and background level were optimized for each single nucleus by human expert. Determination of the parameters was unbiased by drug treatment or the classification of the sub-stage of the S phase of the cell cycle. Estimation of the foci volume was based on an iterative flooding of the maxima in 3D space as described previously in [26]. Classification of γH2AX foci into two classes was based on foci volume and mean fluorescence intensity in raw images, prior to deconvolution. Foci of a volume over 0.25 μm^3^ or a mean intensity over 35 a.u. were classified as “bright” foci. The foci characterized by a lower volume and fluorescence intensity were classified as ‘dim’ (Supplementary Figure 2).

The set of all distances from all γH2AX foci to their nearest replication factory or 53BP1 focus were used to construct a histogram of the distances to the nearest-neighbor. This procedure was repeated for each nucleus, as described previously [16, 20].

Relationship (spatial correlation) of positions of γH2AX foci and replication or 53BP1 foci was characterized over a range of distances using normalized Ripley's K functions (L-functions). The functions were calculated for each nucleus. Detailed description of the algorithm was given in our previous paper [16, 20]. Briefly, value of L = 0 corresponds to random association (proximity) of signals at a given distance, whereas a value L > 0 indicates their non-random association. Thus, L = 1 indicates that the number of pairs of foci (at a given distance) exceeds the respective value obtained for their random distribution by factor of 100%. Difference between the distribution average and 0 was tested at each point (distance) which constitutes the L-function. One should note that the value of L-function may be slightly overestimated at short distances, owing to non-random distribution of nuclear chromatin.

An independent student's *t*-test was used in statistical analysis, using a value of *t* < 0.05 as a measure of significance. The number of nuclei analyzed for each drug treatment in each sub-stage of S phase was at least 18 (the exact numbers are provided in Supplementary Figure 3).

## SUPPLEMENTARY MATERIALS MATERIALS FIGURES


