# Supplementary material for: Low level phosphorylation of histone H2AX on serine 139 (γH2AX) is not associated with DNA double-strand breaks
Source: Oncotarget. 2016 Jul 6;7(31):49574–87. doi: 10.18632/oncotarget.10411 (PMC5226530; doi:10.18632/oncotarget.10411)
Supplement: Supplementary file 1 [file oncotarget-07-49574-s001.pdf]

Low level phosphorylation of histone H2AX on serine 139 (γH2AX) is not associated with DNA double-strand breaks

Supplementary Materials

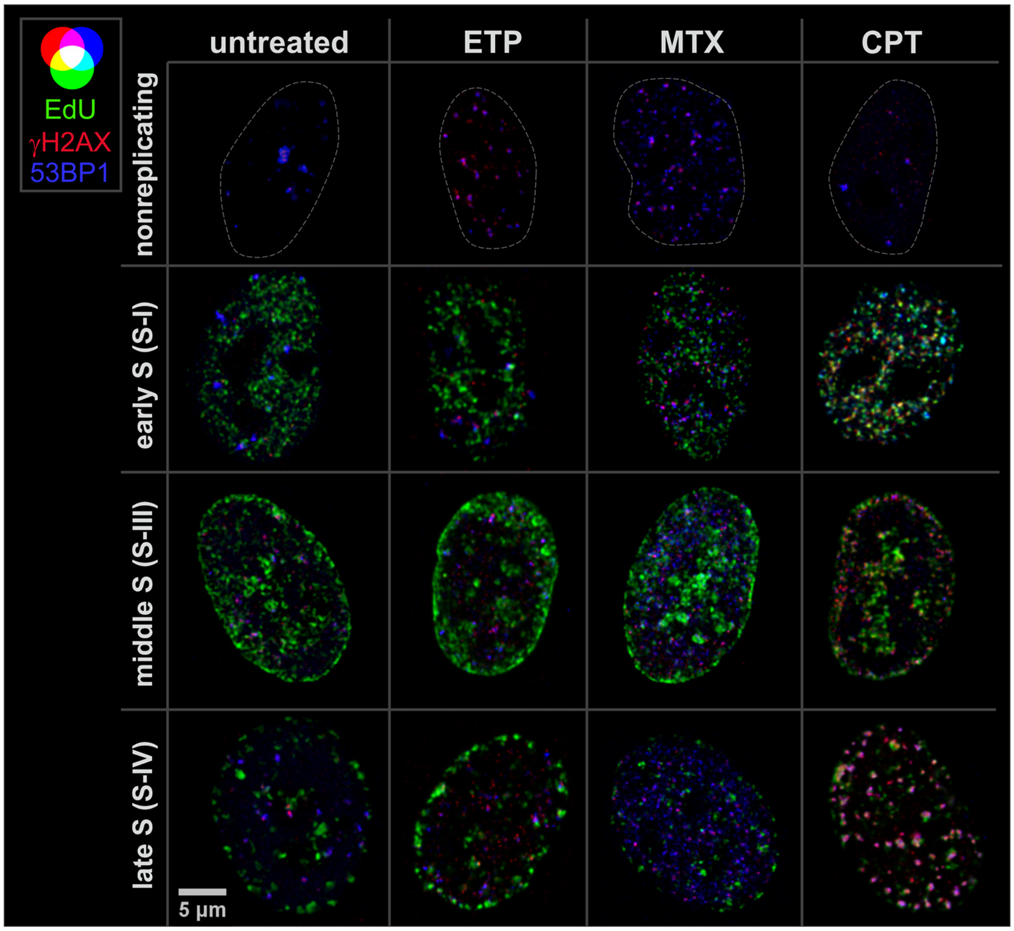

Supplementary Figure S1: Spatial relationships between foci of γH2AX, 53BP1, and EdU. Maximum intensity projection images of central confocal sections (max. z projection of 5 central focal planes) of nuclei immunolabeled for γH2AX and 53BP1, with EdU incorporation sites (replication regions) stained are shown. The samples were prepared according to the scheme in Figure 1A.

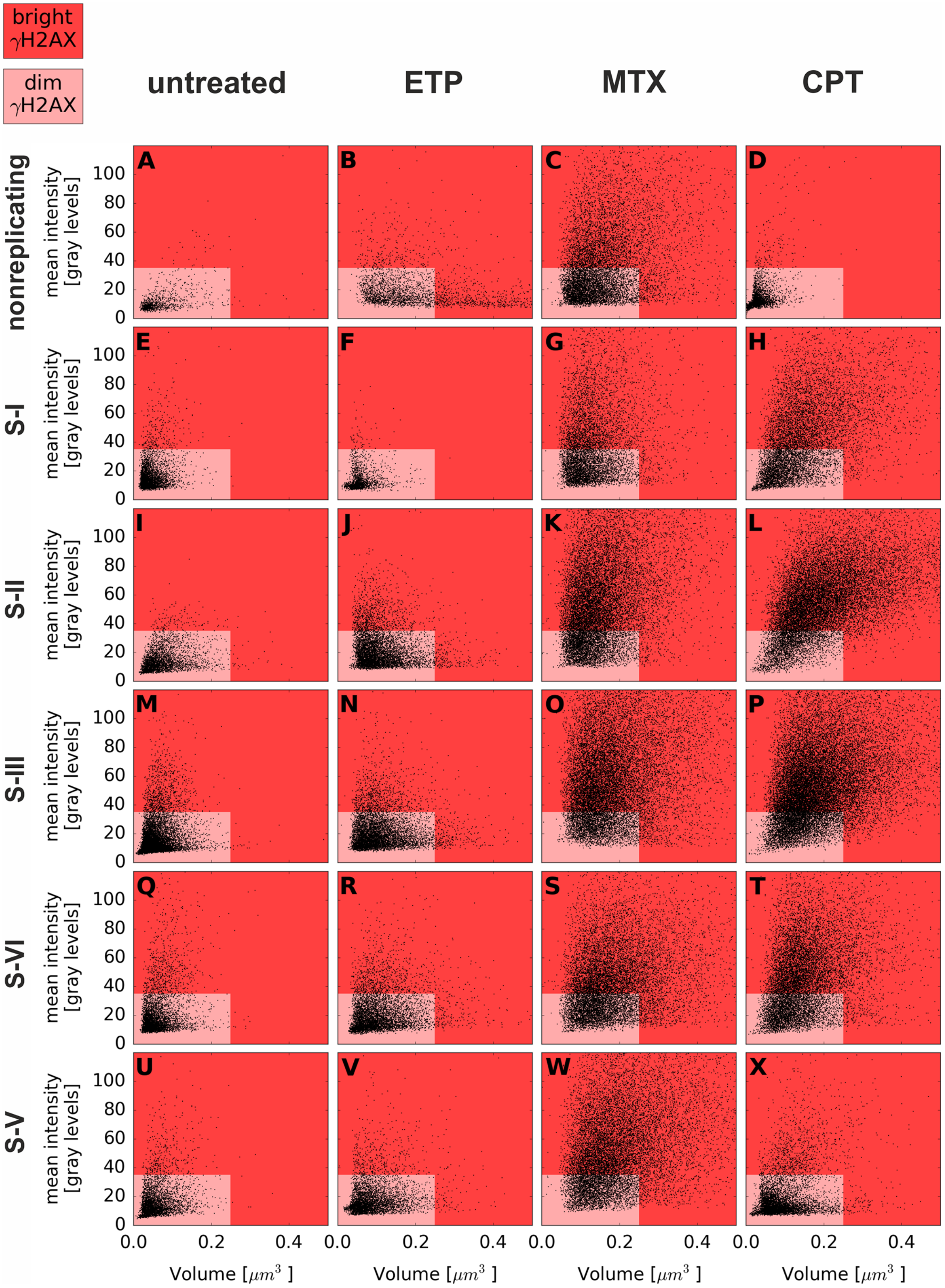

Supplementary Figure S2: Classification of γH2AX foci into two subclasses, "dim" or "bright". Scatterplots depicting the volumes and fluorescence intensities of γH2AX foci. γH2AX "bright" foci are at least 0.25 μm³ in size or at least 35 a.u. bright (red background); γH2AX "dim" foci are at less than 0.25 μm³ in size and less than 35 a.u. bright (pink background).

A **untreated**

	nonrepl.	S-I	S-II	S-III	S-IV	S-V
EdU	0 ± 0	348 ± 92	534 ± 105	732 ± 142	489 ± 126	211 ± 136
bright γH2AX	2 ± 4	10 ± 13	7 ± 9	33 ± 30	31 ± 32	20 ± 22
dim γH2AX	46 ± 27	121 ± 76	138 ± 75	287 ± 134	170 ± 100	194 ± 139
53BP1	16 ± 10	21 ± 13	34 ± 24	40 ± 24	27 ± 22	17 ± 10
number of nuclei	20	27	22	28	22	18

B **ETP**

	nonrepl.	S-I	S-II	S-III	S-IV	S-V
EdU	0 ± 0	279 ± 68	574 ± 88	771 ± 83	578 ± 98	268 ± 148
bright γH2AX	34 ± 43*	2 ± 2*	41 ± 22*	56 ± 39*	53 ± 38	29 ± 26
dim γH2AX	52 ± 40	58 ± 22*	192 ± 47*	275 ± 45	244 ± 57*	222 ± 59
53BP1	15 ± 10	19 ± 12	59 ± 26*	61 ± 41*	35 ± 16	39 ± 26*
number of nuclei	33	28	27	21	20	18

C **MTX**

	nonrepl.	S-I	S-II	S-III	S-IV	S-V
EdU	0 ± 0	315 ± 67	542 ± 89	725 ± 66	495 ± 66	192 ± 124
bright γH2AX	139 ± 84*	167 ± 55*	363 ± 87*	489 ± 86*	334 ± 91*	443 ± 110*
dim γH2AX	153 ± 46*	192 ± 49^	144 ± 73^	127 ± 74^	206 ± 66^	154 ± 88^
53BP1	73 ± 20	95 ± 17*	107 ± 34*	111 ± 50*	108 ± 47*	61 ± 43*
number of nuclei	33	20	28	26	22	22

D **CPT**

	nonrepl.	S-I	S-II	S-III	S-IV	S-V
EdU	0 ± 0	287 ± 70	522 ± 95	663 ± 100	446 ± 74	175 ± 97
bright γH2AX	8 ± 10*	172 ± 97*	476 ± 126*	565 ± 149*	293 ± 112*	40 ± 40*
dim γH2AX	116 ± 99*	132 ± 61^	103 ± 70^	170 ± 163^	180 ± 78^	186 ± 107^
53BP1	15 ± 6*	89 ± 35*	134 ± 74*	249 ± 92*	227 ± 53*	54 ± 49*
number of nuclei	20	29	28	29	24	27

Supplementary Figure S3: The numbers of replication (EdU) and DDR (γH2AX, 53BP) foci in cells exposed to topoisomerase inhibitors - statistical analysis (A–D). A-D The mean values and SD are given; the means that are statistically different for treated and untreated cells are marked with an asterisk (*t*-Student test; 95% confidence limit). Statistical significance was not calculated for cases where the number of dim γH2AX foci was known to be underestimated due to a high foci density and spatially overlapping fluorescence signals (marked with "^").

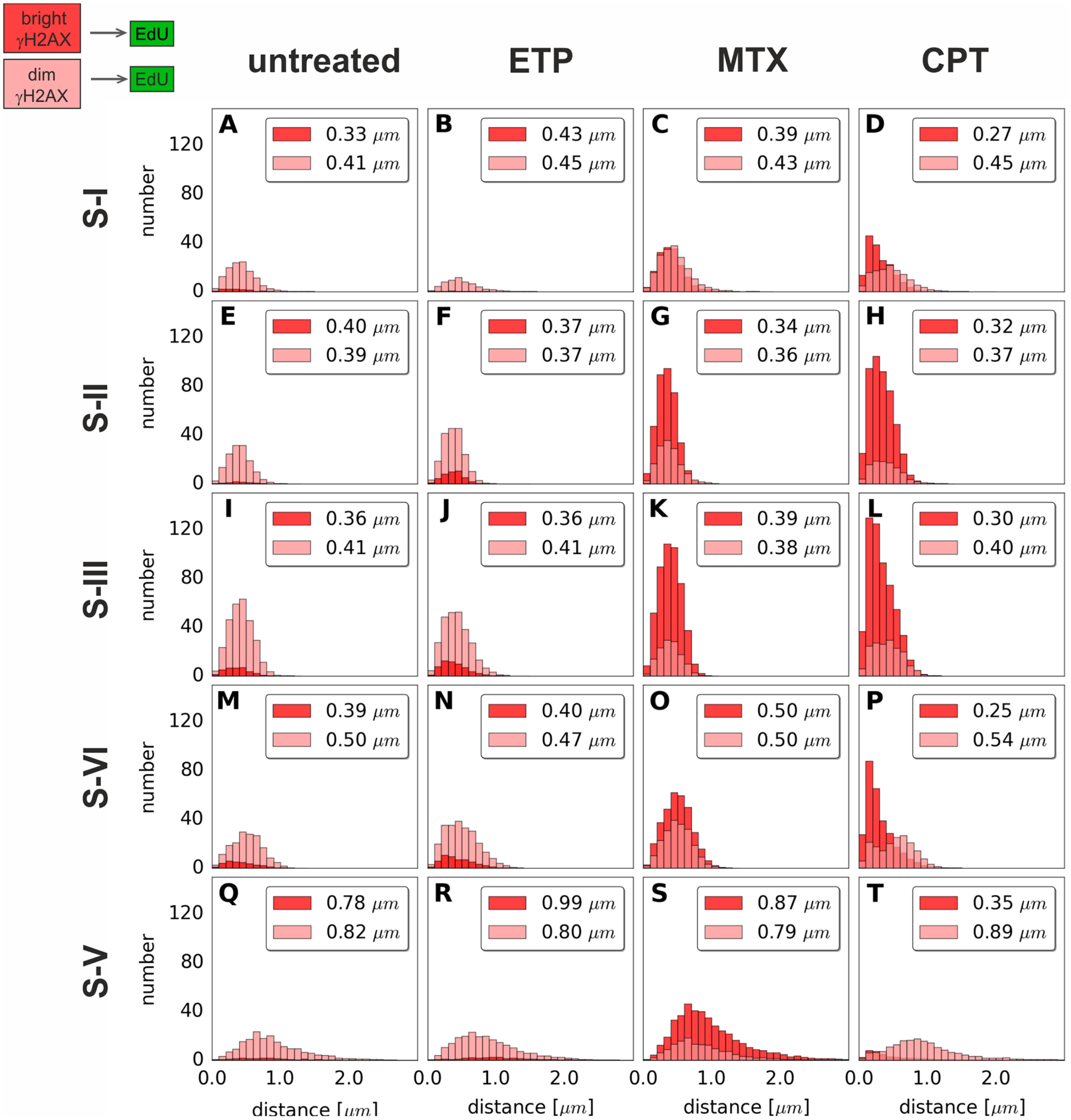

Supplementary Figure S4: Histograms of the distances from γH2AX ("bright" or "dim") to the nearest replication region (EdU) in all sub-stages of S-phase. The median value of the distance is given in the upper right corner of each panel.

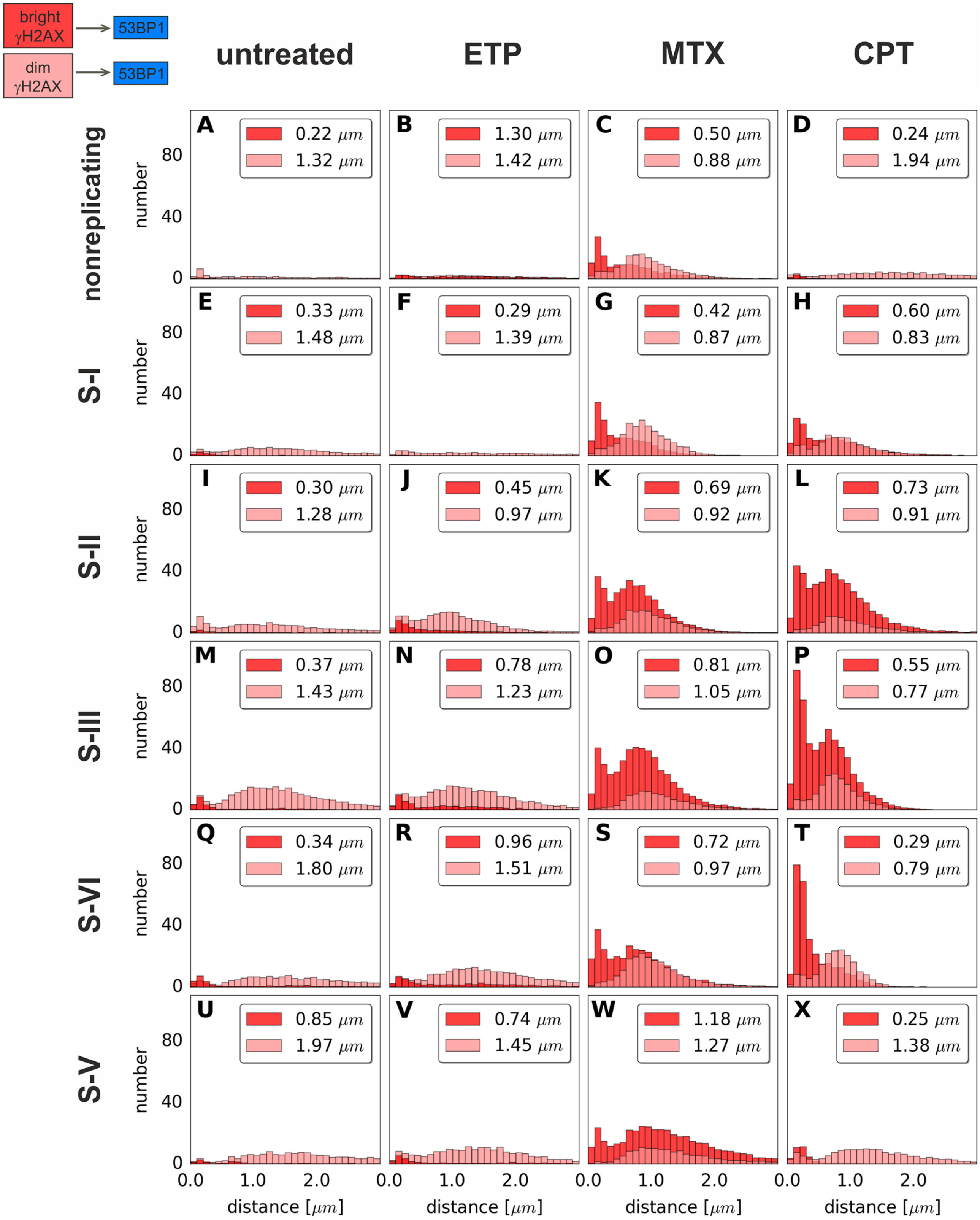

Supplementary Figure S5: Histograms of the distances from 53BP1 foci to the nearest γH2AX ("bright" or "dim") foci, in all sub-stages of S-phase. The median value of the distance is given in the upper right corner of each panel.

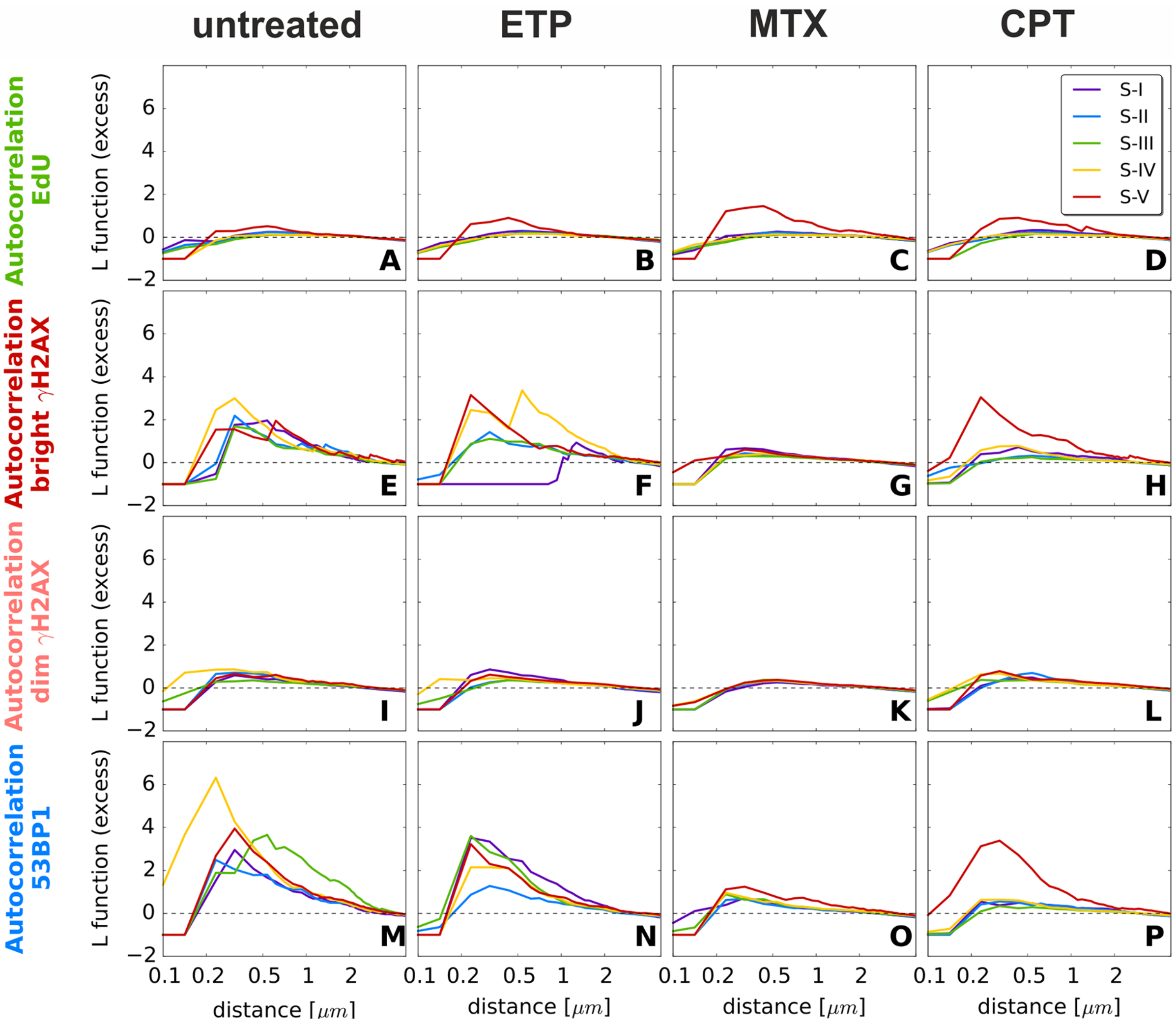

Supplementary Figure S6: Autocorrelation of the patterns of spatial distribution of foci. Replication (**A**–**D**), "bright" γH2AX (**E**–**H**), "dim" γH2AX (**I**–**L**) and 53BP1 (**M**–**P**) foci were studied in nuclei of untreated (A,E,I,M) and ETP-, MTX-, or CPT-treated cells (B,F,J,N; C,G,H,K and D,H,L,P, respectively). The pattern of autocorrelation was analyzed with L-functions (95th percentiles of the single cell dataset).

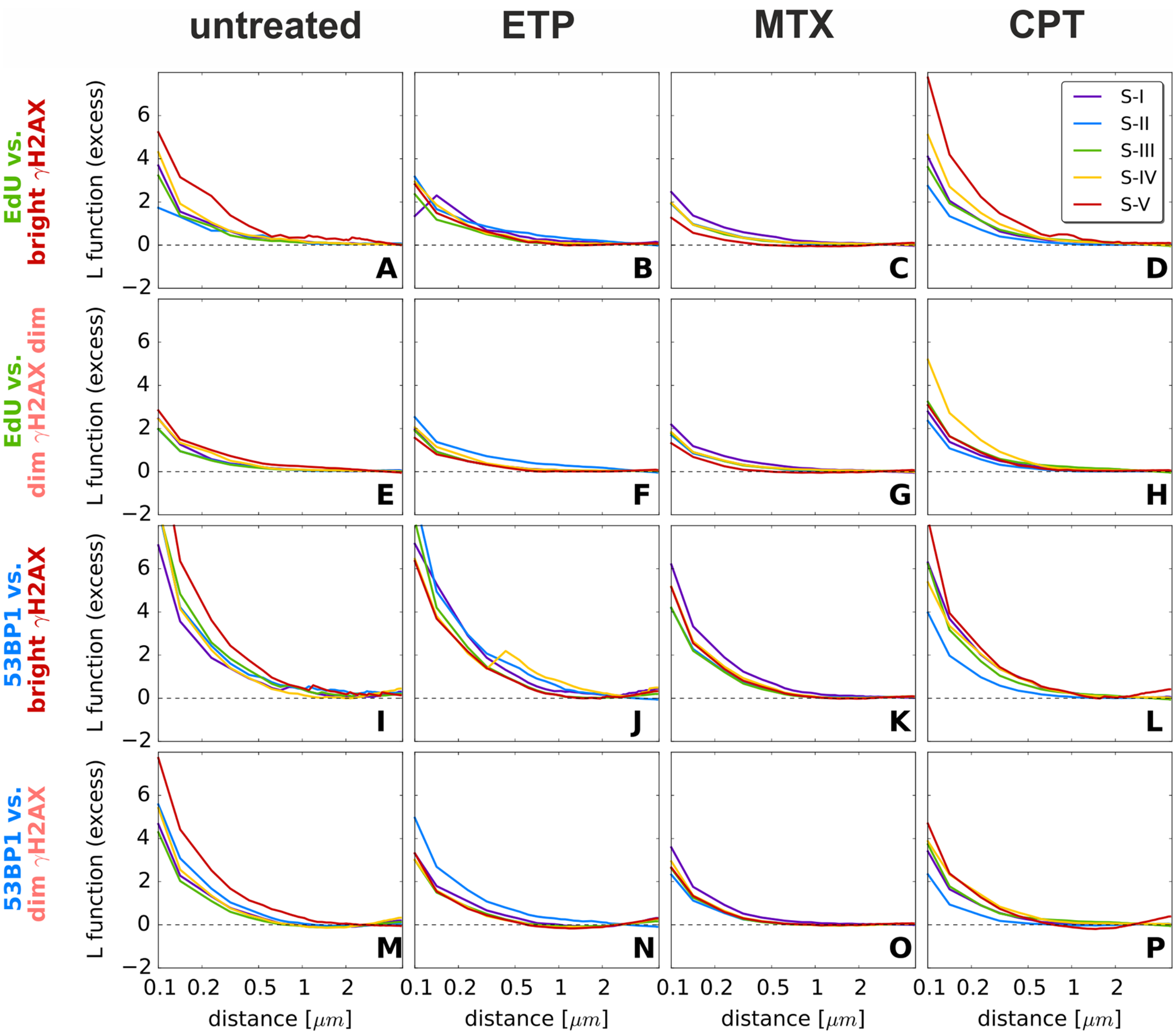

Supplementary Figure S7: Cross-correlation between two patterns of spatial distribution of foci. Patterns of replication (**A**–**H**) or 53BP1 foci (**I**–**P**) and either "bright" (A–D, I–L) or "dim" (E–H, M–P) γH2AX foci were compared using L-functions (95 percentiles). The cross-correlation was analyzed in the nuclei of untreated (A,E,I,M) and ETP- , MTX- or CPT-treated cells (B,F,J,N; C,G,H,K; D,H,L,P, respectively).
